# Narrating the Genetic Landscape of Human Class I Occlusion: A Perspective-Infused Review

**DOI:** 10.3390/jpm13101465

**Published:** 2023-10-06

**Authors:** Iqbal M. Lone, Osayd Zohud, Kareem Midlej, Obaida Awadi, Samir Masarwa, Sebastian Krohn, Christian Kirschneck, Peter Proff, Nezar Watted, Fuad A. Iraqi

**Affiliations:** 1 Department of Clinical Microbiology and Immunology, Sackler Faculty of Medicine, Tel-Aviv University, Tel Aviv 69978, Israel; iqbalzoo84@gmail.com (I.M.L.); osaydzohud@mail.tau.ac.il (O.Z.); kareemmidlej@mail.tau.ac.il (K.M.); 2 Center for Dentistry Research and Aesthetics, Jatt 45911, Israel; awadi.obaida@gmail.com (O.A.); sameer.massarwa@gmail.com (S.M.); nezar.watted@gmx.net (N.W.); 3 Department of Orthodontics, University Hospital of Regensburg, University of Regensburg, 93053 Regensburg, Germany; sebastian.krohn@klinik.uni-regensburg.de (S.K.); christian.kirschneck@klinik.uni-regensburg.de (C.K.); peter.proff@klinik.uni-regensburg.de (P.P.); 4 Department of Orthodontics, Faculty of Dentistry, Arab America University, Jenin 919000, Palestine; 5 Gathering for Prosperity Initiative, Jatt 45911, Israel

**Keywords:** Class I occlusion (CIO), prevalence, quantitative trait loci (QTL) mapping, genome-wide association study (GWAS), epigenetics-wide association study (EWAS), micro and small RNA analysis

## Abstract

This review examines a prevalent condition with multifaceted etiology encompassing genetic, environmental, and oral behavioral factors. It stands as a significant ailment impacting oral functionality, aesthetics, and quality of life. Longitudinal studies indicate that malocclusion in primary dentition may progress to permanent malocclusion. Recognizing and managing malocclusion in primary dentition is gaining prominence. The World Health Organization ranks malocclusions as the third most widespread oral health issue globally. Angle’s classification system is widely used to categorize malocclusions, with Class I occlusion considered the norm. However, its prevalence varies across populations due to genetic and examination disparities. Genetic factors, including variants in genes like MSX1, PAX9, and AXIN2, have been associated with an increased risk of Class I occlusion. This review aims to provide a comprehensive overview of clinical strategies for managing Class I occlusion and consolidate genetic insights from both human and murine populations. Additionally, genomic relationships among craniofacial genes will be assessed in individuals with Class I occlusion, along with a murine model, shedding light on phenotype–genotype associations of clinical relevance. The prevalence of Class I occlusion, its impact, and treatment approaches will be discussed, emphasizing the importance of early intervention. Additionally, the role of RNA alterations in skeletal Class I occlusion will be explored, focusing on variations in expression or structure that influence craniofacial development. Mouse models will be highlighted as crucial tools for investigating mandible size and prognathism and conducting QTL analysis to gain deeper genetic insights. This review amalgamates cellular, molecular, and clinical trait data to unravel correlations between malocclusion and Class I phenotypes.

## 1. Introduction

Malocclusion is an atypical arrangement of teeth or a relationship between dental arches that falls outside the normal range [1]. Malocclusion has a complex etiology that includes genetic, environmental, and hazardous oral behaviors [2]. Malocclusion is a complicated facial skeleton developmental condition affecting the jaws, tongue, and face muscles [3] and stands as one of the three primary illnesses that impair human oral functionality, aesthetics, social interactions, and health-related quality of life [4,5]. Prior longitudinal research has shown that primary dentition malocclusion may lead to permanent dental malocclusion [6,7]. Malocclusion, if left untreated, can progress, ranging from moderate to severe, with variable effects on aesthetics and functionality [8]. Research centered on the early identification and management of malocclusion during primary dentition is becoming increasingly prevalent.

**Figure 1:**
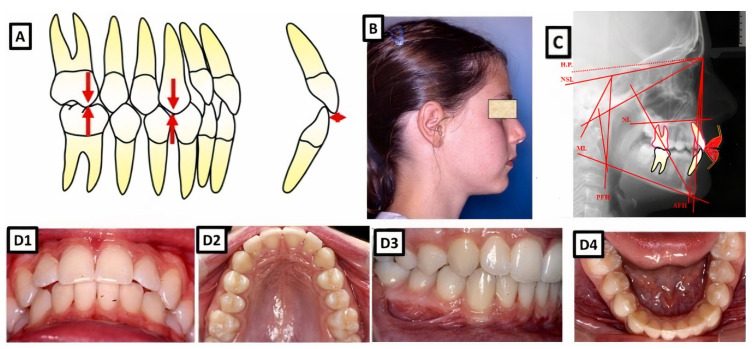
A biometric photo and images of a patient with Class I. In a Class I molar relationship, the mesiobuccal cusp of the maxillary first permanent molar occludes with the buccal groove of the mandibular first molar (**A**). In this definition, the malposition of the teeth, except for the first molars, is not included. (**B**–**D**) show clinical examples, where (**B**) shows extraoral, (**C**) cephalometric, and (**D1**–**D4**) intraoral for dental and skeletal Class I without dentoalveolar malposition of the teeth in both jaws.

According to the World Health Organization, malocclusions are the third most widespread oral health issue, trailing behind dental caries and periodontal diseases [9]. Skeletal abnormalities and malocclusions are diverse disorders that afflict people all over the globe, impairing aesthetics and language function and reducing quality of life [10]. In 1899, Angle established his categorization of occlusions based on the relationship between the buccal groove of the mandibular first permanent molar and the mesiobuccal cusp of the maxillary first permanent molar. Angle Class I occlusion (CIO) is considered the ideal occlusion and is an orthodontic treatment goal for sagittal occlusal anomalies, as shown in Figure 1. This classification is considered one of the most used methods for identifying malpositions of molar relationships [11]. This prevalence varies widely between different populations and ethnicities and is clinically heterogeneous. This variation is likely due to genetic and examination variations in different studies [12,13]. Class I prevalence is considered the most frequent occlusion class globally, ranging from 34.9% to 93.6% in different populations [14,15,16,17]. Like any other malocclusion, Class I occlusions have complicated causes, which are frequently linked to environmental, genetic, and social issues [18]. There is a wide range of published primary research data and reports on Class I occlusion prevalence; the reason for this may be differences in ethnic groups, age groups, registration procedures, and classifications of malocclusions [19].

The genetics and epigenetics of this condition have been the subject of numerous studies in recent years. Research has suggested that Class I occlusion has a complex etiology, with genetic and environmental factors playing a role [20]. Studies have identified several genetic variants associated with an increased occurrence of Class I occlusion, including those in genes involved in craniofacial development such as *MSX*1, *PAX*9, and *AXIN*2 [21]. Additionally, studies have also shown that epigenetic changes, such as DNA methylation and histone modifications, can also play a role in the development of Class I occlusion by affecting the expression of genes involved in craniofacial development [22]. Several genetic variants have been identified as being associated with an increased risk of Class I occlusion. These include single nucleotide polymorphisms (SNPs) in genes involved in craniofacial development. One of them is *MSX1*, a gene that codes for a transcription factor that plays a role in developing the craniofacial skeleton and teeth. Studies have found that SNPs in *MSX1* are associated with an increased risk of Class I occlusion [23]. *EDA* (ectodysplasin A) and *XEDAR* (X-linked ectodermal dysplasia receptor gene) are suggested to be associated with Class I dental-crowding patients [23]. *PAX9* also codes for a transcription factor and is involved in the development of the craniofacial skeleton and teeth. Studies have found that SNPs in PAX9 are associated with an increased risk of Class I occlusion and other dental anomalies, such as hypodontia [24]. The *AXIN2* gene regulates the Wnt signaling pathway, which is essential for craniofacial development. Studies have found that SNPs in *AXIN*2 are associated with an increased risk of Class I occlusion [24].

Other genes that have been identified as associated with Class I occlusion include *ESRRB* [25], *FGF*3 [26], *FGF*4 [27], *FGF*9 [27], *GREM*2 [28], *IRF*6 [29], *JAG*1 [29], *LHX*8 [30], and *TWIST*1 [31]. It’s important to note that most studies on this topic have been conducted on specific populations; the results may not be generalizable to other populations. Additional investigation is required to comprehend the genetic basis of Class I occlusion and how it may vary among different populations.

The primary objective of this review is to provide an overview of the various clinical strategies employed to manage these intricate phenotypes. Additionally, we aim to compile and condense the existing body of knowledge concerning the genetic aspects of Class I occlusion (CIO) in both human and mouse populations.

The report aims to assess genomic relationships among putative craniofacial genes among individuals with Class I occlusion in combination with a murine model. The study characterizes craniofacial skeletal phenotypes in patients with Class I occlusion and generates genetic data on craniofacial genes/loci to identify phenotype–genotype associations of clinical relevance. Several research findings have suggested anterior–posterior and vertical variance in individuals exhibiting Class I occlusion and a certain type of skeletal malocclusion. Prospective research ought to investigate soft-tissue variances to learn more about the genetic basis of skeletal and soft-tissue anomalies in individuals with Class I occlusion. Assessing the genotype–phenotype correlations will help us better comprehend the biological control of postnatal facial development and will guide therapeutic practice to increase the effectiveness of therapy for individuals with occlusion. In addition, we reviewed studies using a mouse model to examine the genetic foundation of mandible dimensions and prognathism.

## 2. Literature Search

We conducted a comprehensive review of peer-reviewed articles in the PubMed and Google Scholar search engines, using the terms “human and mice Class I occlusion”, “genetics of human and mice Class I occlusion”, “QTL mapping and gene associated with human and mice Class I occlusion”, “prevalence of Class I occlusion”, and “treatment of Class I occlusion”.

The literature search was performed between January and April 2023 in the PubMed and Google Scholar search engines, and original articles indexed from early 1990 to January 2023 defining molecular characteristics of skeletal deformities and occlusions were searched for and selected. We found suitable papers based on the inclusion criteria listed below: (1) original research or systematic review, (2) written in English, (3) human Class I occlusion, (4) genetics of human Class I occlusion, (5) QTL analysis and gene linkage with Class I occlusion in humans, and (6) prevalence of Class I occlusion. The exclusion criteria were as follows: (1) transcriptomic or expression analysis without epigenetic/genotyping analysis, (2) articles focused on other diseases in which occlusions were merely mentioned, and (3) articles whose full-text versions were not available to us or that were written in other languages.

Three researchers separately assessed the search record. They reviewed the titles and abstracts and performed a thorough examination of the articles. Any disagreements were addressed by consensus by evaluating either the title/abstract review or the entire manuscript. This method included official reviews of all qualified studies. The selected studies’ quality and possibility of bias risk were appraised alone by the contributors. In cases where disagreements arose among the researchers regarding the inclusion or exclusion of a particular manuscript, a consensus-based approach was employed. The process involved open discussions among the researchers to evaluate the manuscript in question. Any differences in opinion were thoroughly examined, and the researchers worked collaboratively to reach a consensus decision. This consensus-building process was applied to both the title/abstract review phase and the evaluation of the entire manuscript. To maintain transparency and rigor in our methodology, all qualified studies underwent formal reviews, and the selected studies’ quality and potential risk of bias were assessed independently by each contributing researcher. This collaborative and systematic approach ensured that only the most relevant and high-quality studies were included in our review.

## 3. Prevalence of Class I Occlusion

The frequency of Class I occlusion varies by country, gender, and age group. Several researchers have previously reported the incidence of occlusion in Saudi people [32,33,34]. The current study was motivated by little documented information on the prevalence of occlusion features within different cohorts. As a result, having data on occlusion is critical for estimating the total need for therapy. Shaw et al. developed the Index of Treatment Need (IOTN) in the United Kingdom, and due to its straightforwardness and practicality [35], it is broadly acknowledged and regarded as a technique for assessing therapy needs [36,37,38]. Various researchers in various countries have broadly validated the IOTN index’s legitimacy and consistency [35,39,40,41]. Occlusion epidemiological studies not only aid orthodontic therapy strategy but also provide a genuine investigation avenue for identifying environmental and inherited factors that contribute to the genesis of occlusion [42]. In addition, such investigations promise to help with understanding the necessary resources and preventative measures, as well as establishing appropriate healthcare programs. The current study assessed the incidence of occlusion and orthodontic therapy requirements.

The illness burden of occlusion among preschoolers varies significantly worldwide, with incidence rates varying from 26.0% in India [19] to 87.0% in Brazil [43]. Several provinces and cities across mainland China have conducted epidemiological studies on primary dentition occlusion. The Chinese Stomatological Association (CSA) conducted the most recent and most thorough investigation in Chinese children more than two decades ago, revealing a malocclusion rate of 51.84% in Chinese children [44]. Nonetheless, the poll took place in merely 12 regions throughout China. Per our understanding, there is a scarcity of detailed and crucial data about the prevalence of occlusion in deciduous dentition. The current study aims to raise awareness among policymakers and healthcare practitioners on the epidemiological and medical characteristics of occlusion, setting the basis for efficient occlusion avoidance and management in initial dentition.

## 4. Clinical Outcomes of the Phenotype and Clinical Records

The demographic and medical data for the study were obtained from the patient’s orthodontic records, including gender; date of birth; age at treatment initiation; suggested treatment regimen, including extraction and non-extraction of premolars; and length of active orthodontic treatment. To calculate total therapy duration, the starting date was defined as the date of the first molar band placement or first direct bonding, and the completion date was defined as the date of orthodontic retainer delivery.

## 5. Dental Cast Analysis

### 5.1. Mandibular Crowding Assessment

The quantity of mandibular crowding is computed by subtracting the arch perimeter (circumference measured from the mesial of one permanent first molar to its antimere) from the total of the mesiodistal widths of all permanent mandibular teeth except molars [45].

### 5.2. Occlusal Index Computation

The occlusal index is determined using the weighted Peer Assessment Rating (PAR index) established by Ahmad et al. [46], which involves the assessment of five occlusal aspects (posterior occlusion, overjet, overbite, midline, and maxillary tooth displacements) with well-defined measurement criteria (Table 1). The PAR index calculation 30 scores were recorded as follows:

1. Posterior Occlusion

In the original PAR index, posterior occlusion is defined as the area between the contact spot between the canine tooth’s rear and the first permanent molar’s front. The posterior dental link is scored in three spatial planes: anteroposterior, vertical, and transverse deviations, as shown in Table 1. The results are added together and then doubled. Each posterior segment, whether on the right or left side, is captured separately.

2. Overjet

Positive or negative overjet is the horizontal relationship or the distance between the most protruding maxillary central incisor and the opposing mandibular central incisor. Throughout this measurement, the scale is aligned with the occlusal level and radially aligned with the arch axis. The overjet amount was translated to a value via Table 1, after which it was multiplied by 5.

3. Overbite

Overbite is measured in millimeters as a vertical relationship or the distance between the maxillary central incisor and the opposing mandibular central incisor or the degree of open bite, using the tooth with the most significant overlap as a reference. The score was obtained from Table 1, after which it was multiplied by 3.

4. Midline

The score from Table 1 was used to determine the discrepancy of the maxillary midline with the lower central incisors, and it was then multiplied by 3.

5. Maxillary Tooth Displacement

Only in the maxillary anterior region are movements like crowding, spacing, and impacted teeth noted. These occlusal characteristics are noted using the shortest distance between contact points of neighboring teeth parallel to the occlusal plane. The criteria listed in Table 1 are used to convert these measurements into scores, which are then added. When less than 4 mm of space is available for a tooth, it is deemed impacted.

The term “Initial PAR” (PARi) was assigned to the PAR index when calculated from the pre-treatment impressions. Conversely, the term “Final PAR” (PARf) was used when the index was computed based on the post-treatment impressions. The PAR score was calculated by assigning marks to the dental relationships that are intra-arch (such as crowding) and inter-arch (such as overbite, overjet, crossbite, and midline), as well as by using an ordinal scale with an average value of 0. The more significant the value achieved with these indicators, the more serious the malocclusion. Every measurement within the primary and last castings was measured utilizing an electronic instrument.

### 5.3. Assessing Changes in Occlusal Discrepancy

By dividing PARf values by PARi values, the occlusal discrepancy changes brought about by each treatment regimen were computed (PARi—PARF). The index’s numerical decline accounted for occlusal alterations specifically caused by the treatment plan [47,48]. Additionally, the proportion of PAR decrease during therapy (PcPAR) was measured to confirm the degree of recovery compared to the original degree of occlusion [47,48]. The mathematical formula shown below was used to calculate this:

PcPAR = (PARi − PARf)/PARi* 100

### 5.4. Treatment Efficiency (TE) Index

The highest variation in the occlusal index obtained during the shortest duration of treatment is considered efficient. The subsequent equation, where the denominator represents the overall treatment duration, was used to compute this [49].

TE = PcPAR/TIME

### 5.5. Treatment for Class I Occlusion

**Figure 2:**
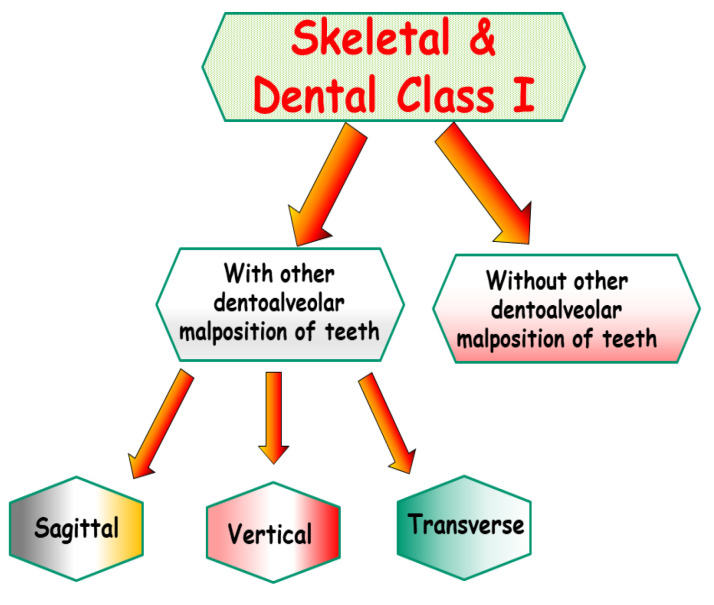
Schematic representation of the possible occurrence of Class I occlusion with or without dentoalveolar malposition of the teeth in all dimensions (sagittal, vertical, and transversal).

**Figure 3:**
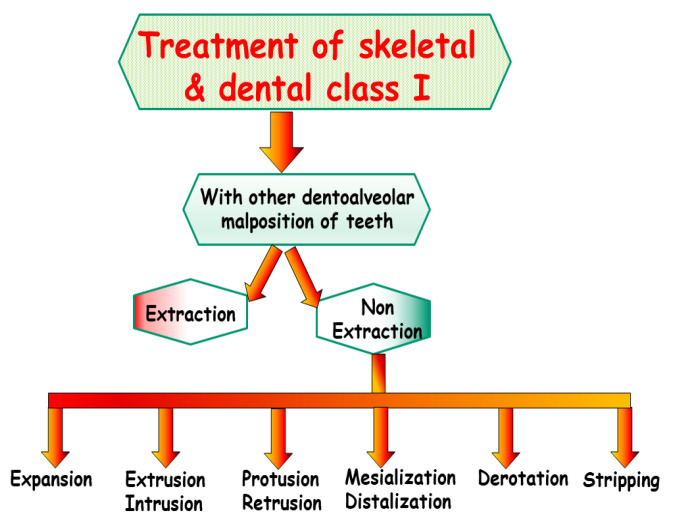
Schematic representation of the treatment options under consideration of the dentofacial aesthetics and function.

**Figure 4:**
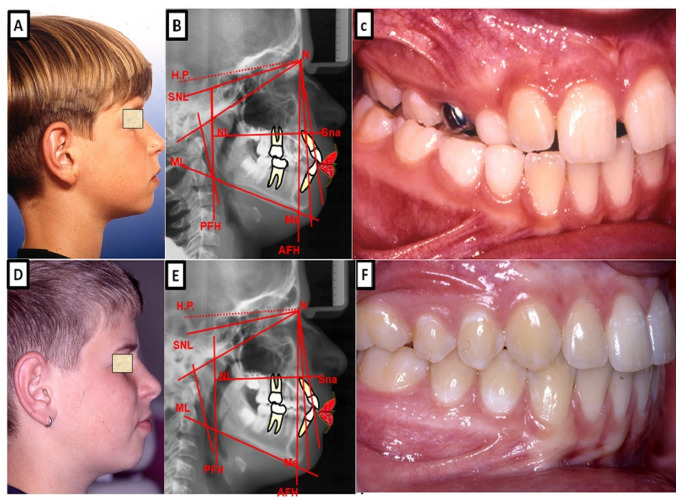
Biometric photo and images of a patient with a Class I occlusion with a transverse problem in the maxilla on the right side (crossbite). The treatment was carried out by transverse up righting of the teeth. (**A**–**C**) are before treatment, and (**D**–**F**) are after treatment.

**Figure 5:**
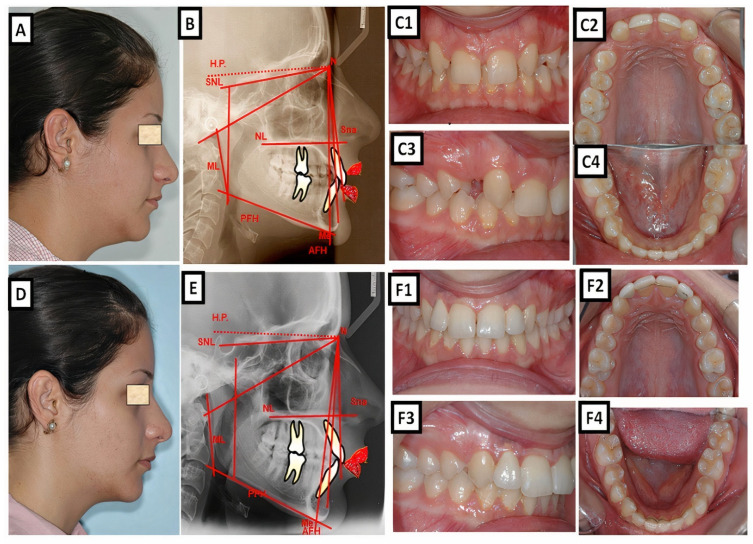
Biometric photo and images of a patient with a Class I occlusion with a vertical problem (deep bite), crowding, and teeth malposition. (**A**–**C**) are before treatment, and (**D**–**F**) are after treatment.

**Figure 6:**
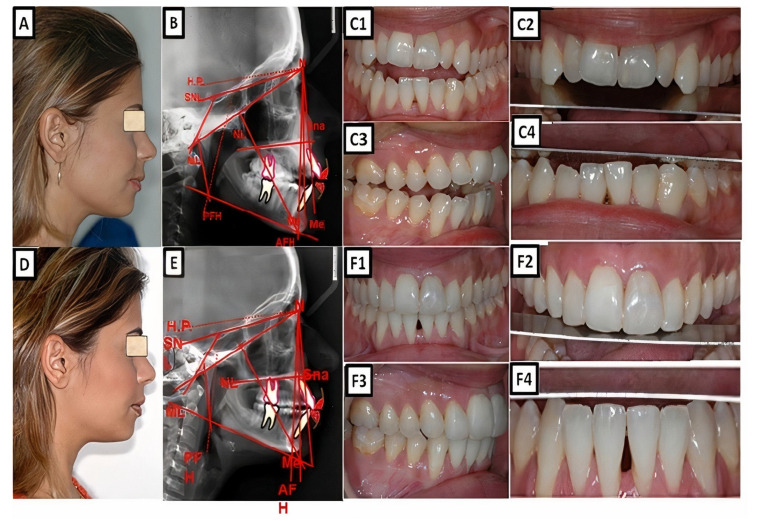
A Abiometric photo and images of a patient with a Class I occlusion with other malpositions of the teeth in the three dimensions: transverse (lateral crossbite), sagittal (increased overjet), vertical (open bite), and crowding. The treatment was performed with a fixed appliance; the front teeth were extruded. (**A**–**C**) show before treatment, and (**D**–**F**) are after treatment.

**Figure 7:**
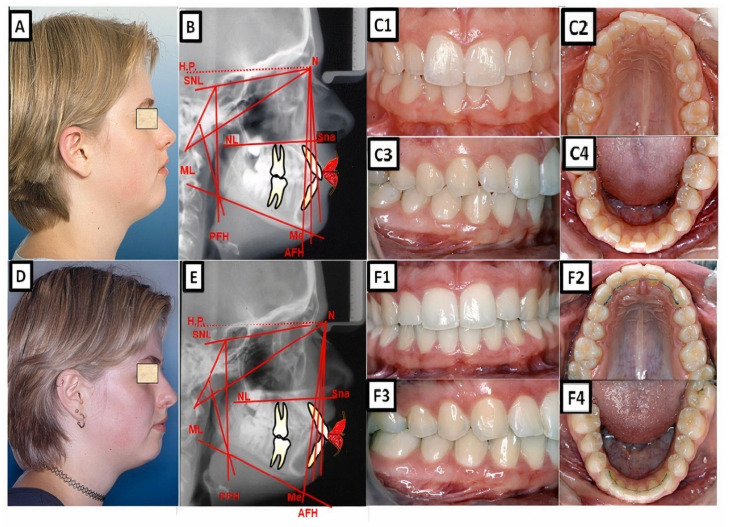
A biometric photo and images of a patient with a Class I occlusion with a dentoalveolar malposition of the teeth and crowding. The treatment was carried out with a fixed appliance. The space was created by approximal enamel reduction. (**A**–**C**) show before treatment, and (**D**–**F**) are after treatment.

**Figure 8:**
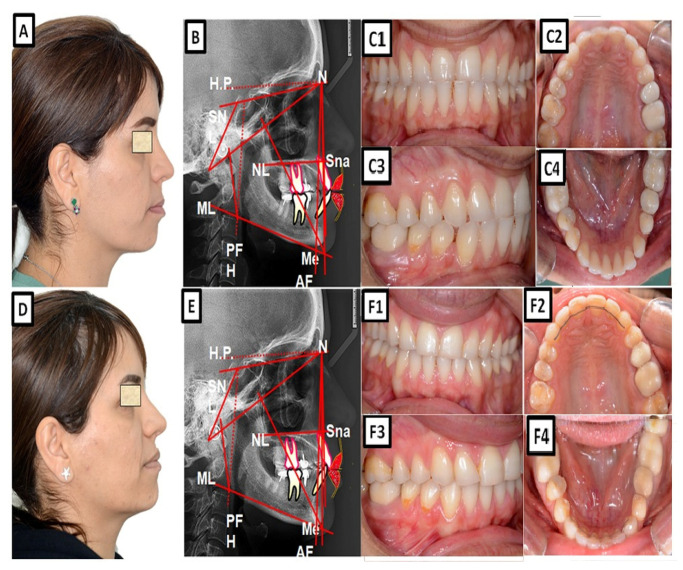
A biometric photo and images of a patient with a Class I occlusion with a sagittal malposition of the frontal teeth (frontal crossbite), crowding, and other teeth malpositions. The frontal crossbite was corrected by protrusion of the upper incisors. (**A**–**C**) show the case before treatment, and (**D**–**F**) are after treatment.

**Figure 9:**
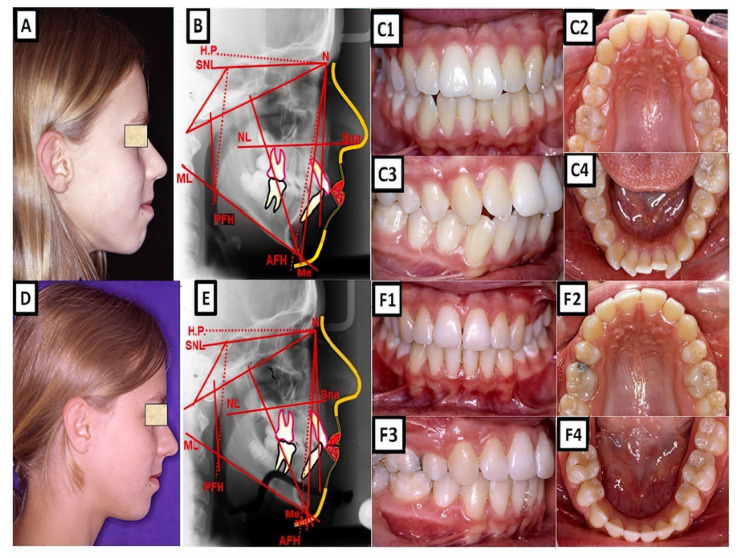
Biometric photo and images of a patient with a Class I occlusion with crowding and other teeth malpositions. The upper and the lower incisors are protruded. For the treatment, four premolars were extracted to retrude the upper and lower incisors and to resolve the crowding. (**A**–**C**) show the case before treatment, and (**D**–**F**) are after treatment.

Since patients often have a favorable soft-tissue environment and harmonious skeletal features, except in bimaxillary cases, Class I occlusions are treated to correct dentoalveolar malpositions of the teeth. Although these dental issues are not specific to Class I occlusion and are observed in other malocclusions, they are described in this study. These dental issues include gaps, tooth malposition (rotation, infraocclusion, supraocclusion, tipping), crowding, impacted teeth, ectopic teeth, crossbites, deep bites, and open bites, as presented in Figure 2. Because the problem is purely dentoalveolar and not skeletal, the treatment will also be dentoalveolar with different devised strategies, as presented in Figure 3, Figure 4, Figure 5, Figure 6, Figure 7, Figure 8 and Figure 9.

### 5.6. Spacing and Crowding

The patient’s skeletal profile, the kind of occlusion, the degree of crowding, the angle of the teeth, the amount of accessible space, and the amount of space required for occlusion correction all play a role in deciding how to close excessive spaces or relieve crowding.

### 5.7. Spacing

Supernumeraries, early tooth loss, microdontia, frenal attachment to the incisive papilla, and congenitally absent teeth can all cause spacing. If possible, the reason for the space should be removed; for instance, a frenectomy is necessary if a median diastema is brought on by a big labial frenum. To assess the existence of supernumeraries in cases with a median diastema, a periapical radiograph is typically also required.

### 5.8. Primary Dentition

Active treatment is not recommended for primary teeth with excessive gaps, based on monitoring.

### 5.9. Mixed Dentition

Depending on the patient’s age, the cause of the spacing, and its severity, spacing can be monitored in mild situations. When the upper canines erupt, the mild divergence and increased space between the upper incisors between the ages of 7 and 12 is considered normal (the “ugly duckling” period). Premature loss of posterior teeth, especially primary second molars, might be a problem during the mixed dentition stage since there is a chance that the permanent first molars may move posteriorly. In these circumstances, the space must be maintained to allow the eruption of permanent successors. The transpalatal arch, lower lingual holding arch, and Nance holding appliance are a few examples of space maintainers. This also aims at preventing a midline shift in the early loss of deciduous canines.

### 5.10. Permanent Dentition

If there is a favorable soft-tissue environment and no serious skeletal abnormalities, excessive gaps can be corrected with clear aligners. With permanent appliances, space closure is also simple to carry out. If the space is caused by tooth loss, one alternative is to use fixed appliances to make enough room, depending on the periodontal health, for an implant or bridge. To obtain the best results in microdontia and peg laterals, a combination of orthodontic and restorative treatment is recommended.

### 5.11. Crowding

Crowding is caused by a size difference between the teeth and dental arches. There are numerous techniques to offer the necessary room for the treatment of crowding, such as arch expander equipment or extraction to gain large spaces and active open-coil springs for acquiring minor spaces.

### 5.12. Primary Dentition

Early-stage crowding results from a lack of primate spaces and indicates that crowding will happen in permanent dentition. The emergence of permanent teeth must also be closely watched, making regular checkups crucial.

### 5.13. Mixed Dentition

Phase I treatment helps to make room in numerous different ways when there is mild to moderate crowding that leads to ectopic eruption or impaction of permanent teeth. The incisors are grouped by partial fixed appliance therapy, or 24, in mild situations. After enough room has been made, a fixed lingual retainer is affixed to the palate of the incisors to stop relapse after the fixed appliances are removed. To maintain the space, space maintainers are bonded, and the eruption of the permanent dentition is tracked. This early intervention aims to stop severe crowding in the permanent dentition, stop ectopic eruptions, and stop the need to remove permanent teeth to make room once growth has stopped. An expander plate, such as a quick maxillary expander or a gradual maxillary expander (quad helix), is used to widen narrow arches before the growth spurt. After enough room has been created with the use of an expander, this form of therapy can be utilized in conjunction with partial braces to align the newly erupted teeth.

### 5.14. Permanent Dentition

A series of aligners can correct mild to moderate crowding when there are no skeletal differences. The use of a detachable appliance can result in the tipping of just one or two teeth. For detachable appliances to work best, high patient compliance is required. Extractions are recommended after the initial growth surge and in situations of extreme congestion. An orthodontist is always the one to decide whether to extract a tooth. The degree of anchorage required heavily influences whether people need space maintainers. Therefore, extractions are always thoughtfully designed with enough anchoring in adults.

### 5.15. RNA Alterations in Skeletal Class I Occlusion

RNA variation refers to variations in the expression or structure of RNA molecules that can affect their function [50]. In the context of skeletal Class I occlusion, RNA variation can refer to variations in the expression or structure of RNA molecules that are involved in the development of the craniofacial skeleton and teeth, which can affect the formation of the jaw and teeth and contribute to the development of skeletal Class I occlusion [29]. There have been several studies that have identified specific RNAs that are altered in skeletal Class I occlusion [51]. However, it is important to note that most of these studies have been conducted on specific populations, and the results may not be generalizable to other populations. In addition, most of the studies focus on specific genes and pathways, and more studies are required to properly comprehend the role of RNA in the formation of skeletal Class I occlusion.

Previous studies have shown that changes in the expression of specific genes can affect the development of the craniofacial skeleton and teeth and may contribute to the development of skeletal Class I occlusion [29]. For example, changes in the expression of genes involved in the Wnt signaling pathway, such as *AXIN*2, can affect craniofacial development and influence the formation of skeletal Class I occlusion [52]. In addition to changes in gene expression, variations in the structure of RNA molecules can also affect their function [53]. For example, variations in the structure of microRNAs (miRNAs), small non-coding RNA molecules that regulate gene expression, have been shown to affect craniofacial development and most likely will contribute to the development of skeletal Class I occlusion [54].

A study found that miR-29 is downregulated in the gingival tissue of individuals with skeletal Class I occlusion and that this downregulation is associated with an increase in the expression of the target genes*COL1A*1 and *MMP*13, which are involved in the regulation of bone remodeling [55]. In addition, miR-31 is downregulated in the gingival tissue of individuals with skeletal Class I occlusion, and this downregulation is associated with an increase in the expression of the target gene*TWIST*1, which is involved in regulating craniofacial development [56]. Further, miR-124 was found to be downregulated in the gingival tissue of individuals with skeletal Class I occlusion, and this downregulation is associated with an increase in the expression of the target gene*Dlx*5, which is involved in the regulation of tooth development [57].

### 5.16. A Mouse Model for Studying Mandible Size, Prognathism, and QTL Analysis

Mouse models have been widely used to study the genetics and epigenetics of skeletal Class I occlusion [31,32,58]. These models allow researchers to manipulate specific genes or environmental factors better to understand their role in the development of this condition. One common approach is to generate mouse models with specific genetic mutations in genes associated with skeletal Class I occlusion. For example, researchers have generated mice with mutations in the *MSX*1, *PAX*9, and *AXIN*2 genes, among others, and observed changes in craniofacial development and tooth formation like those seen in human patients with skeletal Class I occlusion [59,60,61]. Another approach is to use mouse models to study the effects of environmental factors on the development of skeletal Class I occlusion. For example, researchers have used mouse models to study the consequences of maternal nutrition in the formation of the craniofacial bone and teeth and have observed changes in craniofacial development and tooth formation that are similar to those seen in human patients with skeletal Class I occlusion [62,63].

**Figure 10:**
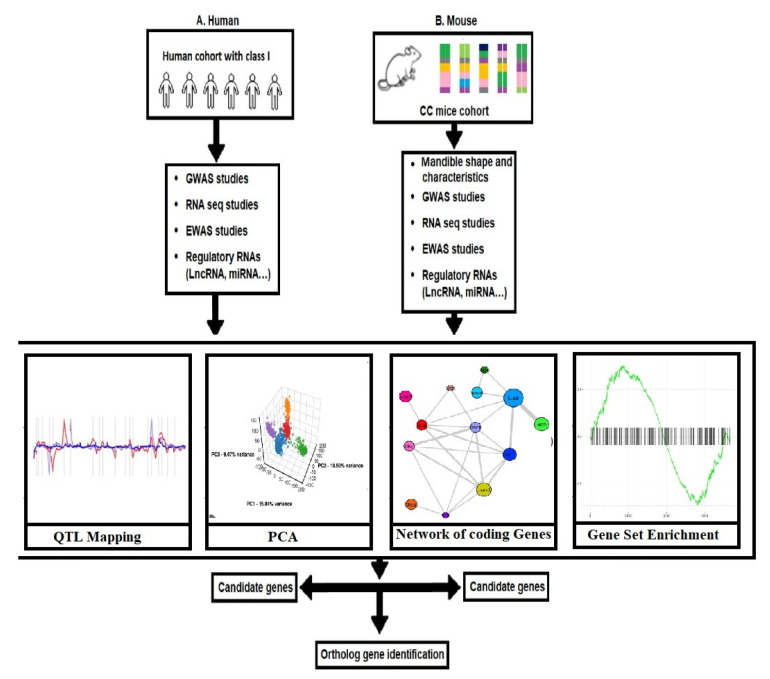
The process for creating systems genetics datasets encompassing cellular, molecular, and clinical trait data is outlined in the workflow. These datasets are amalgamated to facilitate the analysis of correlations between malocclusion and Class I phenotypes. By integrating SNP genotype data, the regulatory genomic regions linked to phenotypic variation are identified. Furthermore, using QTL mapping, specific traits monitored in vitro and in vivo can be pinpointed. Combining these data with subsequent candidate gene association studies conducted in human populations can unveil susceptibility genes linked to the onset of Class I occlusion in individuals.

Mouse models have also been used to study the epigenetic mechanisms involved in developing skeletal Class I occlusion. For example, researchers have used mouse models to study the effects of DNA methylation and histone modifications on gene activity involved in craniofacial development and have observed changes in craniofacial development and tooth formation that are similar to those seen in human patients with skeletal Class I occlusion [64]. Several QTLs for skeletal Class I occlusion have been identified using linkage and association studies in human and animal models. These studies have identified regions of the genome associated with an increased risk of skeletal Class I occlusion and other occlusal traits, such as tooth size and shape and certain craniofacial features [30]. The QTL was identified for skeletal Class I occlusion and other occlusal traits on chromosome 7. In mice, the identified region on chromosome 7 was associated with a significant reduction in overbite, which is a characteristic of skeletal Class I occlusion. Further, a region on chromosome 8 in mice was identified as associated with differences in dental dimensions and morphology. The workflow diagram for generating systems genetics datasets of cellular, molecular, and clinical trait data combined to analyze various correlations between malocclusion and Class I phenotypes is represented in Figure 10.

### 5.17. The Collaborative Cross-Mouse Population—A Potent Resource for Systemic Genetic Analysis of Class I Occlusion

Traditional laboratory mouse strains, on the other hand, possess limited genetic diversity, which makes them less suitable for investigating genetic variations in intricate traits. To overcome this limitation, the collaborative cross (CC) was introduced, generating a novel set of highly genetically diverse recombinant inbred mouse strains. The CC mouse strains were established as a novel resource to enable precise mapping and recognition of the genetic elements responsible for intricate phenotypes, with a specific emphasis on those relevant to human health. The establishment of the collaborative cross genetic reference population (GRP) of mice was driven by the necessity to simulate genetic diversity. This unique genetic reference population (GRP) resource consists of a substantial collection of recombinant inbred (RI) strains. These strains were derived from a genetically diverse selection of eight founding strains, intentionally designed for in-depth analysis of complex traits [65,66,67,68], offering an advantage over any previously documented method [69].

This distinctive resource comprises many recombinant inbred (RI) strains. These strains were generated from a genetically varied pool of eight founder strains, explicitly focusing on facilitating the analysis of complex traits and implying a potency surpassing any previously reported methodologies [70]. The group of eight founder strains demonstrates significant genetic diversity, encompassing five widely used laboratory strains (A/J, C57BL/6J, 129S1/SvImJ, NOD/LtJ, NZO/HiLtJ) and three wild-derived strains (CAST/Ei, PWK/PhJ, WSB/EiJ). This divergence in their phylogenetic origins dramatically contributes to the extensive genetic variation observed within the resulting population of collaborative cross mice.

The CC mouse is a GRP that exhibits a twofold increase in genetic variations, encompassing more than 36 million SNPs. These variations mirror those found within the natural human population, Additionally, it demonstrates a relatively elevated frequency of recombination events in comparison to other mouse sets, with 4.4 million SNPs segregating between the founders [70,71,72]. A recent study involving QTL analysis using the CC population indicated that the mapped interval resolution could potentially be less than 1 Mb [70,71,72,73,74,75,76].

The expansion of the genetic map within the CC population is approximately fourfold, leading to a proportionally enhanced precision in QTL map positioning. Given the inbred origin of all genetic traits, each QTL’s genetic variance is amplified. Moreover, the phenotyping of numerous individuals within each line helps to diminish environmental sources of variance. Compared to conventional F2 mapping populations, this approach significantly multiplies the mapping power of the recombinant inbred line (RIL) set.

CC strains should provide a distinct chance to conduct GWAS and map significant quantitative trait loci (QTL) and subsequently identify candidate genes, as well as mapping modifiers for significant genes associated with Class I traits, while lowering the surrounding obstacles. There is a firm conviction that the substantial genetic diversity present in the founding strains of the CC mouse population offers a robust foundation for uncovering new genetic loci associated with these specific phenotypes. This framework further enables the validation process using mouse knockout genes and conditional knockout methodologies.

### 5.18. The Forthcoming Focus Entails the Creation of an Innovative Model Aimed at Mapping Major and Modifier Genes Linked to Skeletal Class I Malocclusion Using the Collaborative Cross (CC) Model

Systems genetics presents a promising avenue for comprehending the intricate array of biological factors that underlie complex traits within genetically diverse populations. This approach harnesses an array of experimental and statistical techniques to meticulously quantify phenotypes—including transcript, protein, and metabolite levels—within these genetically segregated populations, which exhibit anticipated variations for the traits of interest. Systems genetics investigations have provided an initial holistic perspective of the intricate molecular framework behind complex traits. Such studies are invaluable for pinpointing genes, pathways, and networks that are the foundation for common diseases. In this context, we propose harnessing the capabilities of the CC lines to map genes associated with Class I. Our proposal involves conducting a conventional exploration of candidate genes linked to Class I via GWAS, building upon the successful precedent established by prior publications [65,66,67,68,69,70,71,72,73,74,75,76].

The initial comprehensive exploration of the genetic basis for Class I characteristics has been facilitated through systems genetics analysis, a methodology that aids in identifying genes, signaling pathways, and networks responsible for prevalent disorders. This investigation involves merging data related to cellular, molecular, and clinical aspects to examine the associations among various Class I occlusion phenotypes. By amalgamating SNP genotype data from each CC lineage, regulatory genomic regions implicated in phenotypic variability in both in vitro and in vivo monitored traits are identified. The potential identification of susceptibility genes associated with the onset of Class I occlusion in humans can be achieved by combining data with subsequent investigations in the association of candidate genes in humans.

This experimental design presents the opportunity for parallel in vitro/in vivo screening, bolstered by the development of high-throughput assessment technologies and computational methodologies, leading to a better understanding of how diverse genetic alterations collectively impact the initiation and severity of Class I occlusion. Validated gene–gene interactions and gene–environment networks can be harnessed to inform risk assessment for Class II malocclusion prevention or identify pharmaceutical targets in human systems. Through systems genetics, a comprehensive grasp of the disease’s biology and severity will likely be attained by deciphering the mechanisms of the genetic loci (QTL and genes) uncovered in genome-wide association studies (GWAS) that contribute to susceptibility to Class I occlusion.

Currently, there is extensive molecular research underway concerning regulatory RNAs, encompassing gene expression, DNA methylation, small and microRNA, and long non-coding RNA profiles across a range of diseases. However, to our knowledge, minimal research exists regarding the status of these molecules in the context of skeletal Class II malocclusion. In light of this, we propose that exploring these regulatory RNAs in this condition holds significant promise and will contribute to a more profound comprehension of the molecular underpinnings of the disease.

The workflow diagram presented in Figure 10 outlines the process for generating systems genetics datasets comprising cellular, molecular, and clinical trait data. These datasets are amalgamated to analyze the correlations between Class I phenotypes. The integration of human and mouse approaches, coupled with the application of identification, screening, and exclusion methods, is depicted in the diagram. This systematic approach is a roadmap to facilitate a comprehensive investigation into the intricate mechanisms underlying Class I occlusion.

In conclusion, gaining insight into how the genetic loci (QTL and genes) identified through genome-wide association studies (GWAS) contribute to the susceptibility of Class I phenotypes, in conjunction with the utilization of systems genetics, is poised to enhance our comprehension of both the biology and the nature of the disease.

## 6. Discussion

This study aims to provide a comprehensive understanding of Class I occlusion. The kinds of occlusion in teenage age groups have been described in numerous studies published in the literature from various nations. Though this is the case, comparisons of the findings from these studies are challenging due to differences in the age and size of the study populations and the methodologies used to record occlusal connections. According to reports, the prevalence of malocclusion varies by gender, age, and nation. There havenot been enough investigations to gauge the prevalence [32,33,34]. One of the most straightforward techniques for recording occlusion is calculating the overall frequency of occlusion and the requirement for orthodontic treatment. It is more common to have Angle’s Class I occlusion and less common to have Angle’s Class III occlusion. The results show that Class III malocclusion is the least common malocclusion, with Angle’s Class I and Class II ranking first and second, respectively. According to Al-Emran et al. and Al-Balkhi and Zahrani, Class I, Class II division 1, and Class III malocclusions are the most prevalent in the Saudi population [77,78].

Understanding global epidemiological data aids in establishing priorities for occlusion treatment and the resources needed in terms of work capacity, skills, agility, and materials to be used. To organize the logical planning of orthodontic preventative and therapeutic actions, national public health agencies should be aware of the incidence of occlusion features. Furthermore, evaluations of occlusion prevalence by various groups and geographical regions may reveal the existence of distinct genetic and environmental causes. A precise global picture of the prevalence of occlusion in primary, mixed, and permanent dentitions was produced by this systematic review. Our review found no appreciable variations in male and female occlusion rates, with more than half of children and adolescents worldwide experiencing one type of occlusion. Except for one continent, none of the world’s continents had a reduction in this high incidence to below 50%. The significant number of papers (n = 81) and high level of methodological quality across all included studies provide strong support for the epidemiological relevance of occlusion, according to this review.

The prevalence of occlusion is highest in early childhood during the era of deciduous teeth (54%), and it remains stable during permanent dentition (54%). These prevalence figures show that occlusion is a significant issue for oral health and a financial burden for the families of affected children and public dental health programs. Health policymakers, pediatricians, and dentists should be encouraged to carry out preventive or early diagnosis and develop appropriate treatment strategies because it may be possible to prevent the onset of occlusion from the earliest age (i.e., by avoiding poor oral habits in children) [79,80].

Understanding the type of occlusion and how severe it is will assist in determining whether the group under study requires dental orthodontic treatment and how their oral health is. The current study will also help create customized programs for treating and promoting oral health. According to other observations, refugees are more prone to several ailments, including dental [81]. According to recent studies [82,83,84], refugees have a greater incidence of dental caries and poor oral hygiene than host populations. Untreated dental conditions can cause tooth decay or loss, influencing poor eating patterns and lower quality of life [81]. The most common treatment offered to refugee children, according to a prior study, is extraction, which is a sign of poor oral hygiene and a tendency for refugees to seek dental care late during their sickness, usually for emergency treatment [82,83,84,85]. These results, however, might point to a lack of restorative treatment services due to low funding and a delay in access to dental care [82,83,84,85].

The prevalence of deciduous dentition occlusion is depicted in this meta-analysis in a more precise and thorough manner. According to statistics, various occlusions affect roughly 45.5% of children in mainland China. In addition, significant heterogeneity was found between provinces, which may result from varying criteria, ethnic backgrounds, age ranges, registration processes, or environmental and genetic factors [86,87]. Class I occlusion has the highest predicted occurrence among the two Angle classifications. It is helpful to think about a systematic review of occlusion prevalence among Iranian children that found that poor hygiene and healthcare combined with excessive sugar consumption, which results in caries and the early loss of deciduous teeth, increased the prevalence of Class I occlusion [88]. In addition to genetics, mandibular protrusion and improper feeding practices, like supine nursing, increased the likelihood of occlusion [89]. In Class II malocclusion, a low prevalence rate of 7.97% was noted.

Patients with Class I occlusion who received either four premolar extractions or no treatment should be included in the samples. Since the compatibility of groups for the degree of the initial occlusion will lessen the potential of bias, attention should be on this particular type of occlusion. According to earlier studies, the degree of the initial anteroposterior mismatch influences the length of the treatment and its effectiveness [49,90]. There is less chance of confounding and selection bias because the distribution of sex, age, PARi, and mandibular crowding is consistent between groups. The degree to which genetic variance among individuals can explain variations in their attributes is determined by the degree of heritability [91]. The genetic predisposition to malocclusion susceptibility is supported by a number of data sources. Numerous dental and facial traits, such as mid- and lower facial dimensions, dental spacing, arch dimensions, and Bolton-type tooth size differences, have moderate to high heritability proportions (>60%) documented. On the other hand, overbite (53%) and overjet (28%) have lower heritability, suggesting a larger vulnerability to environmental variables [92,93].

## 7. Conclusions

In this narrative and perspective paper, we have embarked on a comprehensive exploration of Class I occlusion, shedding light on the diverse aspects of this intriguing dental phenomenon. As we delved into the literature, it became evident that although a wealth of research exists on various forms of occlusion, comparing findings across studies remains a challenge. This is primarily due to differences in the age and size of study populations and the methodologies used to document occlusal connections. Furthermore, the prevalence of malocclusion is subject to variations based on factors such as gender, age, and geographical location, leading to complexities in drawing universal conclusions.

Our findings indicated that Angle’s Class I occlusion is the most prevalent, followed by Class II, whereas Class III malocclusion is the least common. Understanding global epidemiological data is crucial to establishing priorities in occlusion treatment and the allocation of resources, including workforce, skills, agility, and materials. National public health agencies must be informed about the prevalence of occlusion characteristics to strategically plan orthodontic preventative and therapeutic interventions. Furthermore, analyzing occlusion prevalence across diverse demographic groups and regions may reveal distinct genetic and environmental factors that contribute to its occurrence.

Of particular interest is the observation that occlusion prevalence peaks during early childhood with deciduous teeth (54%) and remains stable during permanent dentition (54%). These prevalence figures underscore the critical role of occlusion in oral health and its economic implications for families and public health programs. This insight calls for proactive measures, such as preventive strategies and early diagnosis, to address occlusion-related issues, including children’s oral habits.

Additionally, our study highlights the challenges faced by vulnerable populations, such as refugees, who often experience higher rates of dental conditions. These disparities necessitate increased attention from health policymakers, pediatricians, and dentists to develop targeted strategies for prevention, diagnosis, and treatment.

This narrative review and perspective paper offers a multifaceted exploration of Class I occlusion, drawing from diverse sources and perspectives. By unraveling the complexities of occlusion, we aim to contribute to the broader discourse on oral health, inform public health policies, and inspire future research endeavors in this fascinating field.

## Figures and Tables

**Table 1:** Criteria applied to score each component of the Peer Assessment Rating (PAR index).

Occlusal Relationships	Discrepancy	Score	Weight	
Anteroposterior	Good interdigitation—Class I, II, or III	0	2	PosteriorOcclusion
	Less than half of premolar width	1		
	Half of premolar width	2		
Vertical	No discrepancy in intercuspation	0	2	
	Posterior open bite on at least two teeth greater than 2 mm	1		
Transverse	No crossbite	0	2	
	Crossbite tendency	1		
	Single tooth in crossbite	2		
	More than one tooth in crossbite	3		
	More than one tooth in scissor bite	4		
Positive	0–3 mm	0	5	Overtjet
	3.1–5 mm	1		
	5.1–7 mm	2		
	7.1–9 mm	3		
	Greater than 9 mm	4		
Negative	No discrepancy	0	5	
	One or more teeth edgetoedge	1		
	One single tooth in crossbite	2		
	Two teeth in crossbite	3		
	More than two teeth in crossbite	4		
Negative	No open bite	0	3	Overbite
	Open bite less than and equal to 1 mm	1		
	Open bite 1.1–2 mm	2		
	Open bite 2.1–3 mm	3		
	Open bite greater than or equal to 4 mm	4		
Positive	Less than or equal to 1/3 coverage of lower incisor	0	3	
	Greater than 1/3 but less than 2/3 coverage of lower incisor	1		
	Greater than 2/3 coverage of lower incisor	2		
	Greater than or equal to full coverage of lower incisor	3		
Crowding	0–1 mm displacement	0	1	Displacement
	1.1–2 mm displacement	1		
	2.1–4 mm displacement	2		
Spacing Impaction	4.1–8 mm displacement	3		
	Greater than 8 mm	4		
	Impacted teeth	5		
Midline	Coincident and up to 1/4 lower incisor width	0	3	
	Deviated 1/4 to 1/2 lower incisor width	1		
	Deviated more than 1/2 lower incisor width	2		

## Data Availability

Not applicable.
